# Advances in Heterotrophic Nitrification–Aerobic Denitrification: Physiological Characteristics, Environmental Responses, and Nitrogen Metabolic Pathways

**DOI:** 10.3390/microorganisms14102174

**Published:** 2026-09-26

**Authors:** Yuanyuan Yang, Junkang Wu, Zhaoqian Jing

**Affiliations:** 1 Department of Water Science and Technology, College of Civil Engineering, Nanjing Forestry University, No. 159 Longpan Road, Nanjing 210037, China; 2 Jiangsu Carbon Sequestration Materials and Structural Technology of Bamboo & Wood Research Center, Nanjing Forestry University, Nanjing 210037, China

**Keywords:** heterotrophic nitrification, aerobic denitrification, biological nitrogen removal, environmental responses, metabolic mechanism

## Abstract

Heterotrophic nitrification–aerobic denitrification (HNAD) bacteria enable nitrogen removal under aerobic conditions and have attracted increasing attention in wastewater treatment. Although numerous studies have enriched HNAD strains and explored their nitrogen removal performance, the environmental responses and metabolic mechanisms underlying nitrogen transformation remain insufficiently evaluated. In this review, the ecological distribution and physiological characteristics of HNAD bacteria are summarized, followed by a systematic elaboration of the effects of nitrogen sources, carbon sources, C/N ratio, and environmental factors (e.g., temperature, pH, dissolved oxygen and salinity) on nitrogen removal and transformation performance. In addition, nitrogen depletion from the aqueous phase is likely to reflect both conversion to gaseous nitrogen products and assimilatory uptake into biomass, which may account for a non-negligible proportion of the apparent nitrogen removal in some strains. Accordingly, the nitrogen transformation mechanisms, encompassing nitrite/nitrate-mediated pathways, proposed hydroxylamine-associated pathways, and nitrogen assimilation, are critically examined, with attention to the strength and limitations of the available evidence. Furthermore, the regulatory roles of intracellular redox balance and electron allocation in mediating the interplay among nitrification, denitrification, and cellular respiration are addressed. Finally, the key challenges and future prospects of HNAD research and applications are highlighted. This review provides mechanistic insights into the metabolic versatility of HNAD bacteria and supports the development and evaluation of nitrogen removal strategies in advanced biological nitrogen removal systems.

## 1. Introduction

In recent years, water pollution caused by excessive nitrogen emissions, including municipal sewage, industrial wastewater, livestock breeding wastewater, and agricultural non-point source pollution, has emerged as a critical environmental concern and attracted increasing attention worldwide [1,2]. Among inorganic nitrogen pollutants, ammonia nitrogen warrants particular attention due to its greater acute toxicity to aquatic organisms compared with nitrate. Its toxicity mainly depends on the equilibrium between ammonium (NH_4_^+^) and the toxic free ammonia (NH_3_), which varies with pH and temperature [3]. Effective ammonium removal is therefore particularly important for mitigating the immediate ecological risks of nitrogen-rich effluents. Biological nitrogen removal (BNR) has been widely applied due to the advantages of high nitrogen removal efficiency, low cost and low secondary pollution when compared to the physical-chemical nitrogen removal processes [4,5]. In the mainstream wastewater treatment plants (WWTPs), BNR processes mainly include aerobic nitrification and anoxic denitrification, where ammonia is oxidized to nitrate by autotrophic nitrifiers under aerobic conditions and nitrate is subsequently reduced to nitrogen gas by heterotrophic microorganisms under anoxic conditions [6].

Nowadays, several new nitrogen biotransformation processes have emerged to overcome the deficiencies in the conventional BNR systems, mainly including simultaneous nitrification and denitrification (SND), endogenous denitrification (ED), anaerobic ammonium oxidation (Anammox), partial nitrification/denitrification (PN/D), sulfur autotrophic denitrification (SADN), hydrogen autotrophic denitrification (HD) and Heterotrophic nitrification–aerobic denitrification (HNAD) [7]. Among these processes, HNAD enables nitrification and denitrification to occur within a single organism/system under aerobic conditions [7,8], which provides opportunities to integrate nitrogen and organic matter removal in a single aerated system and therefore eliminates the need for separate oxic and anoxic treatment stages [8]. Heterotrophic nitrification (HN) generally refers to the oxidation of reduced nitrogen compounds, including ammonium and certain organic nitrogen compounds, to nitrite and/or nitrate by heterotrophic microorganisms [9], and this process may not be directly coupled to energy conservation for driving ammonia oxidation. Aerobic denitrification (AD), by contrast, is the reduction of nitrate or nitrite to gaseous nitrogen products under aerobic conditions [10], where organic carbon may serve as an electron donor, an energy source, and a carbon source for biomass synthesis [11]. At the enzymatic level, ammonia monooxygenase (AMO) and hydroxylamine oxidase (HAO) activities have been reported in individual HNAD strains [12], while enzyme purification and characterization in *Thiosphaera pantotropha* identified a hydroxylamine oxidase enzyme distinct from those of autotrophic nitrifiers [13]. Aerobic denitrification mainly involves nitrate reductases (Nar/Nap), nitrite reductases (NirK/NirS), nitric oxide reductase (Nor), and nitrous oxide reductase (NosZ), and the activity is linked to carbon availability and cellular redox balance through electron partitioning between oxygen respiration and nitrogen oxide reduction [14,15]. However, the involvement of canonical nitrification enzymes or genes cannot be assumed for all HNAD strains. For example, genomic analysis of *Pseudomonas stutzeri* UFV5 identified denitrification genes but failed to identify genes encoding the canonical autotrophic nitrification enzymes [16], which suggests the potential existence of alternative ammonia-oxidizing functional genes or pathways in HNAD strains.

The occurrence of HNAD has overcome the limitations of the conventional BNR process. Compared with autotrophic nitrifiers, HNAD bacteria have been reported to grow faster, exhibit greater adaptability to environmental fluctuations, and utilize organic substrates more efficiently [17,18]. By combining heterotrophic nitrification and aerobic denitrification, HNAD enables simultaneous nitrogen and COD removal in a single aerobic system [19], although the performance strongly depends on bacterial strain, nitrogen substrate, carbon source and availability, and environmental conditions, particularly dissolved oxygen (DO) [16,19]. Compared with anaerobic ammonium removal (e.g., anammox) systems, HNAD offers distinct process advantages, particularly for wastewater containing organic carbon [20]. For example, HNAD enables simultaneous nitrification and denitrification, providing simpler operation, faster start-up, and greater adaptability to environmental fluctuations, as well as enhanced robustness toward organic carbon loading [21,22]. These characteristics offer potential advantages for treating wastewaters containing both ammonium and biodegradable organic matter and may reduce reactor footprint and operational complexity.

Currently, various HNAD bacteria have been reported in multiple environments, including landfill leachate [23,24], wastewater treatment plants [25,26], activated sludge [17], soil [27], rivers [28], marine aquaculture systems [29], and coking wastewater [30], with reported isolates belonging to genera such as *Acinetobacter*, *Bacillus*, *Cuprobacter*, *Halomonas*, *Klebsiella*, *Marinobacter*, *Pseudomonas*, and *Photobacterium* [31]. However, these HNAD strains exhibit distinct responses to environmental conditions and often require specific physiological settings for optimal performance. In addition, the colonization potential and competitive fitness of individual strains within complex microbial communities remain poorly understood. Recent studies have suggested that different HNAD bacteria may adopt distinct nitrogen metabolism strategies, reflecting the complexity of HNAD pathways. Although several representative metabolic pathways of HNAD have been proposed, the exact nitrogen transformation routes and their regulatory mechanisms remain to be fully elucidated. Accordingly, a systematic understanding of bacteria-mediated HNAD processes is still needed, particularly regarding the nitrogen transformation pathways and the underlying metabolic mechanisms.

Previous reviews have summarized HAND strain diversity, environmental influences, metabolic pathways, and key enzymes [32], as well as the coupling of organic carbon metabolism and nitrogen conversion through electron transport [8]. Building on these syntheses, integrating strain-specific physiological responses with experimental evidence for nitrogen transformation pathways is essential to explain variation in reported HNAD performance. Recent findings have also highlighted the need to reassess proposed nitrogen removal pathways: isotope-based and transcriptomic analyses of *Alcaligenes faecalis* identified hydroxylamine as the primary nitrification product and detected no denitrification under the conditions tested [10]. An updated synthesis is therefore needed to link environmental responses to strain-specific metabolic evidence and distinguish shared physiological features from mechanisms established only in individual isolates.

This review focuses on HNAD bacterial isolates characterized in pure culture and comprehensively summarizes their distribution and physiological characteristics. The key environmental factors affecting the performance of HNAD bacteria are also systematically evaluated. Furthermore, the HNAD metabolic mechanisms are examined in terms of electron transfer and nitrogen transformation. Building on previous reviews, particular attention is given to the relationships among strain characteristics, cultivation conditions, and proposed nitrogen transformation pathways. Finally, critical challenges and future prospects for HNAD research and application are highlighted. This review aims to provide a clear framework for understanding the physiological characteristics and metabolic mechanisms of HNAD strains, which could thereby support the development and optimization of efficient biological nitrogen removal processes in wastewater treatment systems.

## 2. Distribution and Enrichment of HNAD Bacteria

### 2.1. The Discovery and Progress

In 1983, Robertson and Kuenen reported the isolation of *Thiosphaera pantotropha*, later reclassified as *Paracoccus pantotrophus*, from a desulfurizing and denitrifying effluent-treatment system [33]. A subsequent study demonstrated that this bacterium could simultaneously perform heterotrophic nitrification and aerobic denitrification in aerobic chemostat cultures [34]. Since then, extensive research has focused on the isolation of various HNAD strains and evaluation of their nitrogen removal performance under different environmental conditions [31,35]. Table 1 summarizes 22 taxonomic genera of representative HNAD strains isolated in pure culture, together with their isolation sources and characterization methods. These strains have been experimentally shown to exhibit both HN and AD activities to remove ammonium, nitrite, and nitrate effectively.

HNAD strains have been isolated from a wide range of environments, including activated sludge, wastewater, soil, and freshwater lakes [32], as shown in Figure 1. Organic carbon utilization and aerobic nitrogen transformation provide a physiological basis for HNAD bacteria to exploit habitats containing suitable carbon and nitrogen substrates [58]. In some isolates, tolerance to a wide range of salinity and pH may further facilitate their occurrence or persistence in particular habitats [40]. However, enrichment and screening inherently select for strains capable of both growth and nitrogen removal under the selected substrate and cultivation conditions [59]. Therefore, the isolates obtained may not fully represent the natural HNAD community.

Moreover, numerous studies have reported HNAD-associated genera in specific engineered treatment systems. For example, in marine aquaculture wastewater treatment systems, *Bacillus* and *Halomonas* were identified as keystone genera associated with HNAD activity [60]. In wastewater treatment plants, the putative HNAD-associated genera *Denitratisoma*, *Dechloromonas*, and *Comamonas* showed their highest combined relative abundance in the A/O process, accounting for 7.69% of the microbial community [61]. In addition, *Acinetobacter* was reported to dominate the putative HNAD-associated community in MBR sewage treatment systems [62]. These community profiles suggest the occurrence of potentially relevant taxa. However, the contribution of these taxa to HNAD still requires functional verification. The reported isolation sources in Table 1 provide a basis for selecting samples for further enrichment, purification, and characterization.

### 2.2. The Enrichment and Purification

In existing studies, HNAD bacteria are generally isolated through selective enrichment and subsequent purification [63,64]. Most previously reported HNAD strains have been enriched under aerobic conditions in chemically defined media, where ammonium, nitrite, or nitrate serves as the sole/mixture nitrogen source, with an organic carbon source provided to support heterotrophic metabolism [65,66,67]. These conditions impose selective pressure by favoring microorganisms capable of nitrogen transformation under simultaneous aerobic, heterotrophic conditions. Nevertheless, evidence of nitrogen removal obtained solely during enrichment is insufficient to establish an HNAD phenotype, as the disappearance of inorganic nitrogen may also reflect assimilation or other nitrogen-transforming processes [68]. In addition, HNAD activity may be attributed to a single strain or to community-level cooperation [69,70]. Community-level nitrogen removal alone is, therefore, insufficient to establish that individual members perform both heterotrophic nitrification and aerobic denitrification, nor does it indicate which members contribute to these processes. After purification, strain-specific assessment should distinguish ammonium assimilation from heterotrophic nitrification and evaluate nitrate and/or nitrite reduction under aerobic conditions [27]. Analysis of nitrogen transformation products and nitrogen mass balances [71,72], supplemented by enzyme assays or genetic evidence, can further help substantiate the proposed pathways.

A wide range of HNAD strains affiliated with genera such as *Alcaligenes*, *Pseudomonas*, and *Acinetobacter* have been successfully isolated and purified as shown in Table 1. For example, Li et al. enriched HNAD bacteria from activated sludge in an MBR wastewater treatment system and isolated 53 HNAD strains, among which Acinetobacter was the dominant genus [62]. Previous studies have demonstrated that enrichment under specific selection pressures (e.g., high salinity, high nitrogen loading and low C/N ratio) may favor the isolation of HNAD strains capable of growth and nitrogen removal under the selected conditions. For instance, enrichment under saline conditions yielded halotolerant strains such as *Halomonas venusta* TJPU05, which achieved over 80% removal of multiple nitrogen species (NH_4_^+^, NO_3_^−^ and NO_2_^−^) at high salinity (8%) [73]. *Alcaligenes faecalis* TF-1, isolated from landfill leachate with a strategy of gradual increase in nitrogen loading, was reported to tolerate an extremely high concentration of NH_4_^+^-N at 2000 mg N L^−1^ and achieved 94.6% NH_4_^+^-N removal under optimized conditions [74]. Similarly, *Acinetobacter* sp. WZ-1, obtained under low C/N enrichment conditions, removed 47–88% of NH_4_^+^-N at C/N ratios of 4–8 [75]. Collectively, these examples suggest that tailoring enrichment conditions to the target environment may help obtain HNAD strains with the desired physiological and nitrogen-removal characteristics. The microbial responses of HNAD strains to specific environmental factors are further discussed in Section 4.

## 3. Physiological Characteristics of HNAD Bacteria

### 3.1. Morphological Diversity of Reported Isolates

Bacteria capable of HNAD belong to diverse taxonomic groups, and this taxonomic diversity is usually reflected in varying morphological features. Among the reported isolates, rod-shaped and short-rod forms predominate, although coccoid and pleomorphic forms have also been documented, which is dependent on taxonomic affiliation and cultivation conditions [64]. In addition, reported cells typically measure 0.5–1.0 μm in width and 1.0–3.0 μm in length [27].

### 3.2. Kinetic Parameters

The maximum specific growth rate ($\mu_{\max}$) and apparent half-saturation coefficient ($K_{s}$) are commonly used to describe the growth kinetics of HNAD bacteria. In a Monod growth model, $\mu_{\max}$ represents the maximum specific growth rate under the specified conditions, whereas $K_{s}$ is the concentration of the growth-limiting substrate at which the growth rate reaches half of $\mu_{\max}$ [76]. $K_{s}$ is an apparent, model-dependent coefficient specific to the substrate, process, and experimental conditions, rather than a direct measure of intrinsic biochemical affinity [77]. Apparent $K_{s}$ values estimated for ammonium, nitrite, nitrate, or other growth-limiting substrates are therefore not directly interchangeable. Reported $\mu_{\max}$ values for the HNAD strains summarized in Table 2 range from approximately 0.18 to 0.60 h^−1^, with variation reflecting both strain characteristics and cultivation conditions. Comparisons of kinetic parameters require sufficiently comparable substrates, processes, and experimental conditions. These growth parameters help characterize growth potential for the specific HNAD strains under their respective cultivation conditions, but may not establish a competitive advantage in the mixed communities.

### 3.3. Nitrogen Source Selectivity

HNAD bacteria can utilize diverse inorganic nitrogen sources, including NH_4_^+^-N, NO_2_^−^-N and NO_3_^−^-N under aerobic conditions [32]. However, substrate preferences vary among strains and can influence growth and nitrogen removal performance (Table 3) [55]. Generally, NH_4_^+^ is more readily utilized than NO_2_^−^ or NO_3_^−^ [83], although substrate preference may vary depending on microbial species and environmental conditions.

When NH_4_^+^-N was provided as the sole nitrogen source, most HNAD strains often exhibited comparable or improved nitrogen removal performance and growth behavior when compared with the oxidized nitrogen sources (e.g., NO_2_^−^-N and NO_3_^−^-N), although this response was highly strain-dependent. This preference may partly reflect that NH_4_^+^ can be directly utilized in HN or directly assimilated into biomass, whereas oxidized nitrogen forms require additional reduction steps before incorporation into cellular biomass [89]. For example, *Pseudomonas stutzeri* YG-24 exhibited average NH_4_^+^-N, NO_2_^−^-N and NO_3_^−^-N removal rate of 8.8, 7.5 and 7.7 mg N L^−1^ h^−1^, respectively [90]. In addition, ammonium has also been demonstrated to be incorporated into biomass through assimilation pathways such as the glutamine synthetase-glutamate synthase (GS-GOGAT) system, thereby contributing to cellular nitrogen incorporation during the HNAD process [91]. Moreover, nitrogen balance analysis using isotope tracing method showed that 62.4% of the converted ammonium was assimilated into biomass in *Corynebacterium glutamicum* WN1, while 33.79%, 0.13%, and 0.04% were recovered as N_2_-N, N_2_O-N, and NH_2_OH, respectively [21]. These findings indicate that assimilation contributes substantially to ammonium removal. The direct assimilation of ammonium may therefore partly explain the favorable growth and efficient substrate removal observed when ammonium is used as the sole nitrogen source. However, ammonium disappearance alone does not demonstrate heterotrophic nitrification. Distinguishing assimilatory uptake from other nitrogen transformations requires additional evidence, such as nitrogen-product measurements, isotope tracing, and nitrogen mass balances, depending on the experimental system.

NO_3_^−^-N utilization may involve enzyme induction and sequential reduction. During complete aerobic denitrification, nitrate is reduced through nitrite, NO, and N_2_O to N_2_ by the corresponding reductases [92]. This process requires organic electron donors, making carbon availability and the C/N ratio important factors affecting nitrate removal [93]. An adaptation phase may occur when nitrate is supplied as the sole nitrogen source [94]. For example, *Acinetobacter* sp. HA2 exhibited a lag phase of approximately 8 h when switching from NH_4_^+^-N to NO_3_^−^-N as the sole nitrogen source, which was attributed to the induction of nitrate reductase synthesis [67].

NO_2_^−^-N, an intermediate in both nitrification and denitrification, can exert concentration-dependent effects on HNAD activity. At relatively low concentrations, NO_2_^−^-N can be rapidly transformed and may support denitrification [95,96]. At elevated concentrations, nitrite and its protonated form, free nitrous acid (FNA), may inhibit nitrogen-metabolizing enzyme activity and compromise cellular integrity, with their relative importance depending on pH and other cultivation conditions [97,98]. Nevertheless, some HNAD strains retain nitrogen-removal activity at elevated nitrite concentrations. For instance, *Alcaligenes faecalis* WT14 exhibited rapid growth and nearly complete removal of an initial NO_2_^−^-N concentration of 100 mg N L^−1^ within 24 h when nitrite was supplied as the sole nitrogen source at an initial pH of 7.0 and 30 °C [99]. Collectively, these findings indicate that some specific HNAD strains can efficiently utilize nitrite even at elevated concentrations; however, nitrite tolerance and inhibition should be evaluated in terms of both NO_2_^−^-N concentration and pH-dependent FNA formation rather than nitrite concentration alone.

## 4. Environmental Responses of HNAD Bacteria

### 4.1. Effects of Carbon Source and C/N Ratio

Organic carbon availability is an important factor influencing nitrogen removal by HNAD bacteria, as organic carbon can provide energy, reducing power, and carbon skeletons for cell synthesis [8,100]. Under heterotrophic nitrification conditions, organic carbon supports biomass synthesis and cellular metabolism, but may not directly drive ammonia oxidation as an electron donor [34,45]. During aerobic denitrification, organic substrates can serve as electron donors for nitrate or nitrite reduction, support energy generation, and provide carbon for biomass synthesis [33,101,102]. Nevertheless, the extent of dependence on external organic carbon may vary among strains and experimental conditions [103,104]. Generally, carbon source type and C/N ratio jointly influence HNAD performance by modulating central carbon metabolism and the generation and partitioning of reducing equivalents, as schematically illustrated in Figure 2.

#### 4.1.1. Influence of Carbon Source on Heterotrophic Nitrification

Heterotrophic nitrification in HNAD bacteria is closely linked to intracellular carbon metabolism. The reducing power (e.g., nicotinamide adenine dinucleotide, NADH) and ATP, could be generated via glycolysis and the tricarboxylic acid (TCA) cycle during ammonium and carbon metabolism, which supports cellular growth and maintains redox balance [94]. The optimal carbon sources for different HNAD bacteria and the nitrogen removal rates were shown in Table 4.

Existing research have shown that small organic acid salts generally outperform carbohydrates in promoting ammonium removal. For instance, in *Pseudomonas* sp. B-1, sodium succinate resulted in the highest ammonium removal efficiency (ARE) value of 57.5%, whereas glucose yielded only 16.1% [114]. Similar trends were observed in *Acinetobacter tandoii* MZ-5 [115] and *Halomonas campislis* ha3 [72], which exhibited enhanced ammonium removal when low-molecular-weight organic acids were used as carbon sources. This metabolic advantage can be attributed to the direct incorporation of substrates such as succinate and acetate into the TCA cycle, bypassing glycolysis and reducing metabolic burden, which ultimately leads to enhanced energy generation and stable redox conditions [23,44,116,117]. In contrast, carbohydrates typically undergo glycolysis and, in some cases, extracellular hydrolysis before entering central metabolism [23,118]. The additional enzyme-mediated reactions not only delay electron release but also divert carbon flux toward biomass synthesis [119]. Consequently, carbon source type regulates carbon flux distribution and electron availability within central metabolism, thereby influencing ammonium oxidation efficiency. Furthermore, different carbon sources might occupy distinct positions in cellular metabolic networks, influencing redox balance and enzyme regulation [120]. Accordingly, carbon sources that sustain continuous ATP generation and stabilize NADH/NAD^+^ balance are more favorable for efficient nitrogen transformation.

Overall, carbon sources can significantly influence HN through their effects on carbon flux, energy supply, and redox balance. In addition, low-molecular-weight organic acid salts usually favor ammonium removal despite of the different preferences among individual strains.

#### 4.1.2. Influence of Carbon Source on Aerobic Denitrification

Unlike HN, AD is a reductive pathway that directly depends on intracellular reducing equivalents [96]. During aerobic denitrification, reducing equivalents generated through organic carbon metabolism can supply electrons for nitrate and nitrite reduction via electron-transfer components [15,101]. Therefore, carbon sources with high reducing power yield are expected to enhance denitrification efficiency.

Readily biodegradable carbon sources (e.g., acetate, citrate, succinate and other organic acid salts) have been reported to typically enhance aerobic denitrification rates by rapidly generating reducing power and promoting electron transfer to NO_3_^−^/NO_2_^−^ reductases [121]. For example, strain *Acinetobacter* Y1 exhibited significantly higher nitrogen removal efficiencies when utilizing sodium pyruvate, citrate, or acetate when compared with sucrose and glucose [122], which is similar to the strain *Acinetobacter* sp. ND7 [70]. In addition, carbon metabolism-derived ATP plays a crucial role in cellular energy metabolism and the activation of denitrification processes. In *Acinetobacter johnsonii* WGX-9, sodium acetate enhanced ATP synthesis and membrane transport activity, achieving an NO_3_^−^ removal efficiencies around 86.6% at a rate of 0.2 mg N L^−1^h^−1^ [123]. A similar effect was also observed in *Acinetobacter* sp. TAC-1 [124]. It can therefore be inferred that carbon sources not only supply electrons but also influence enzyme synthesis through cellular energy status, thereby supporting efficient denitrification performance.

#### 4.1.3. Effect of C/N Ratio on Nitrogen Removal Performance

Moderate-to-high C/N ratios (commonly 8–15) have been frequently reported to favor HNAD performance [31]. As shown in Table 4, increased carbon availability with increasing ratios generally enhances NADH and ATP production, thereby sustaining simultaneous oxygen respiration and aerobic denitrification [8,105]. Under carbon-limited conditions, the supply of reducing equivalents became insufficient to support both oxygen respiration and aerobic denitrification pathways, which usually resulted in incomplete denitrification or reduced ammonium oxidation efficiency.

Nevertheless, several HNAD strains maintain high nitrogen removal efficiencies even at low C/N ratios (≤3–4), including *Janthinobacterium svalbardensis* F19 (2) [125], *Acinetobacter* sp. Y16 (2) [126], and *Acinetobacter haerbinensis* HITLi7^T^ (3–4) [127]. These findings suggest that high carbon availability is not a universal prerequisite for efficient HNAD metabolism and that carbon requirements may vary with strain-specific differences in intracellular electron allocation and nitrogen transformation strategies [8,75]. In terms of electron and energy allocation, carbon-sensitive strains such as *Acinetobacter junii* ZHG-1 were strongly inhibited under carbon limitation due to decreased NADH/NAD^+^ ratios and reduced electron transport system activity (ETSA) [105]. In contrast, *Bacillus thuringiensis* WXN-23 maintained a high specific nitrogen removal rate under a relatively low C/N ratio (5.9), possibly by preferentially allocating available reducing equivalents toward nitrogen reduction rather than biomass synthesis [128]. The mechanistic basis underlying strain-specific electron allocation patterns and nitrogen transformation pathways in HNAD bacteria is further discussed in Section 5.2.

Overall, C/N dependency should not be interpreted solely as a preference for higher organic loading. It more fundamentally reflects the balance between electron generation and electron consumption within the cell [105]. When electron production exceeds respiratory demand, nitrogen removal is enhanced. While electron supply is limiting, oxygen respiration may outcompete denitrification [96]. In addition, strain-specific differences in this balance account for the observed variability in optimal C/N ratios. Understanding of the distinct metabolic patterns could provide new insights into the design of low-carbon nitrogen removal systems.

### 4.2. Effects of Environmental Factors

Environmental parameters can significantly influence enzyme activity and the allocation of intracellular reducing equivalents [129]. This section evaluated how key environmental factors, including temperature, pH, and DO, influence metabolic flux and electron transfer. The environmental characteristics and nitrogen removal performance of representative HNAD bacteria reported in previous studies were summarized in Table 5.

#### 4.2.1. Temperature

Temperature strongly regulates the metabolic activity and nitrogen removal performance of HNAD bacteria. The majority of documented HNAD strains exhibited a clear mesophilic preference, with optimal nitrogen removal typically occurring at 25–37 °C [135]. For example, *Delftia tsuruhatensis* SDU2 achieved the highest ammonia removal efficiency (93.7%) at 30 °C, whereas both cell growth and nitrogen removal decreased significantly at lower (20 °C) or higher (>35 °C) temperatures [51]. Similarly, *Pseudomonas putida* ZN1 showed negligible growth and denitrification activity at 10 °C or above 40 °C, whereas nitrogen removal efficiency exceeded 90% between 15 °C and 30 °C [88]. In general, nitrogen removal performance increased with temperature up to the optimum range (typically 25–37 °C), mainly owing to enhanced enzyme activity and microbial growth rates [136].

Although most HNAD bacteria exhibited mesophilic characteristics, growing evidence suggests that specific HNAD strains possess remarkable cold-tolerance capabilities and maintain efficient nitrogen removal performance. Cold-tolerant strains such as *Janthinobacterium* sp. J1-1 were able to achieve high removal of NH_4_^+^-N (around 89.2%) and NO_3_^−^-N (around 68.2%) at 8 °C [53]. In addition, psychrotolerant strains such as *Bacillus simplex* H-b and *Priestia aryabhattai* KX-3 have been reported to exhibit high ammonium removal efficiencies of 82.2% and 92.5%, respectively, even at 10 °C [101,137]. Strain-specific physiological and biochemical responses may contribute to cold tolerance in HNAD bacteria. In *Acinetobacter calcoaceticus* TY1, transcriptomic and biochemical analyses identified changes in fatty-acid composition, extracellular polysaccharide production, and antioxidant responses under cold stress [138]. These observations support a proposed role for membrane-related and oxidative-stress responses in maintaining cellular function at low temperatures. In *Pseudomonas indoloxydans* YY-1, increased ATP levels and extracellular polysaccharide content were reported following a temperature decrease, together with changes in the expression of genes involved in energy metabolism [139]. These measurements provide experimental evidence of cold-associated physiological responses. However, the contributions of individual responses to sustained nitrogen removal require further validation and should not be assumed to apply to all HNAD strains.

It is worth noting that thermophilic HNAD bacteria have also been identified. For instance, *Anoxybacillus contaminans* HA was reported to survive at temperatures up to 60 °C and exhibits optimal denitrification activity at 55 °C, with NH_4_^+^-N and NO_3_^−^-N removal efficiencies exceeding 70% [140]. However, the molecular mechanisms underlying temperature tolerance in HNAD bacteria remain insufficiently understood and require further investigation. The coexistence of psychrotolerant and thermophilic HNAD bacteria indicates substantial physiological plasticity in their nitrogen transformation pathways.

#### 4.2.2. pH Value

pH is another critical environmental factor regulating the growth and nitrogen transformation activity of HNAD bacteria. Current studies have shown that HNAD bacteria can survive within a relatively wide pH range of 4–10 [141]. However, such viability does not necessarily imply comparable growth or nitrogen transformation activity throughout this range. In general, most strains exhibit optimal growth and nitrogen removal efficiency under neutral to slightly alkaline conditions [142,143]. For example, the ammonium removal efficiency of *Pseudomonas mosselii* K17 exceeded 85% within an initial pH range of 6–9 and reached approximately 95% at pH 7 [133].

Despite this general preference, several acid-tolerant HNAD bacteria have also been reported. For example, *Acinetobacter* sp. JR1 isolated from acidic pharmaceutical wastewater achieved a high ammonia removal efficiency of 97.9% at an initial pH of 4.5 [144]. In addition, *Pseudomonas fragi* EH-H1 was found to exhibit complete ammonium removal within 20 h at an initial pH of 6.2 [145]. These findings demonstrate that certain HNAD strains can retain ammonium removal activity under acidic initial conditions, although they do not by themselves establish sustained performance under fluctuating pH conditions.

The reported pH optima are generally condition- and strain-dependent, and multiple mechanisms may contribute to the pH response. Firstly, pH can influence membrane potential and proton gradients, thereby affecting ATP generation and nutrient transport [146,147]. In addition, extreme pH conditions can alter enzyme conformation and catalytic activity, thereby affecting nitrogen transformation activity [148]. Moreover, microorganisms may maintain intracellular pH homeostasis through proton transport systems and stress-response regulation [146]. These mechanisms may support pH tolerance, but do not necessarily preserve growth or nitrogen removal efficiency. Therefore, the observed pH optimum may be shaped by multiple interacting factors, including nitrogen speciation, membrane energetics, enzyme activity, substrate transport, and pH-dependent free-ammonia toxicity, with their relative contributions likely depending on the strain and cultivation conditions [149]. These mechanisms should be viewed as possible contributors to pH responses and tolerance, rather than as a complete explanation for the observed pH optimum of a given strain.

#### 4.2.3. Dissolved Oxygen

DO is acknowledged to serve as a critical regulatory switch governing the HNAD process by directly mediating the cellular redox environment and intracellular electron distribution. Appropriate DO levels are essential to sustain the metabolic coupling between HN and AD; they provide sufficient oxygen for aerobic respiration and ammonia oxidation while ensuring that nitrogen oxides (NO_2_^−^ and NO_3_^−^) can still efficiently compete for electrons [150]. Driven by their rapid heterotrophic metabolism, many HNAD bacteria can quickly consume oxygen to create localized microaerobic niches within cellular aggregates, thereby enabling the concurrent operation of HN and AD pathways [151].

Changes in oxygen availability can affect nitrogen removal by altering the balance between aerobic respiration and nitrogen transformation. Low DO may limit oxygen-dependent ammonium oxidation and respiratory energy generation [152]. In contrast, high DO may favor oxygen respiration and reduce the availability of electrons for denitrification, particularly when the supply of reducing equivalents is limited [105,153]. The resulting effects on ammonium removal and denitrification depend on the strain, carbon availability, and cultivation conditions. DO should therefore be evaluated together with these factors when identifying suitable conditions for nitrogen removal.

Recent studies have underscored that the balance between oxygen utilization and electron allocation is maintained through diverse strain-specific physiological characteristics. For example, *Pseudomonas* sp. AND-42 exhibited the highest ammonium removal performance at a DO concentration of approximately 3.0 mg·L^−1^, whereas higher DO levels (>8.0 mg·L^−1^) resulted in a decline in NH4^+^-N removal efficiency, indicating the existence of a strain-specific oxygen optimum for coupled nitrogen conversion [154]. Similarly, *Pseudomonas putida* ZN1 achieved maximum ammonium removal (97.5%) at a shaking speed of 120 rpm corresponding to a DO concentration of 4.67 mg·L^−1^, while either increasing or decreasing oxygen availability significantly reduced nitrogen removal performance [88]. In contrast, *Pseudomonas stutzeri* KTB exhibited a preference for relatively high oxygen availability and achieved complete nitrogen removal within 18 h at a shaking speed of 160 rpm [81]. Such tolerance to elevated oxygen availability may be associated with strong self-aggregation and rapid oxygen consumption, which have been reported to facilitate the formation of localized microaerobic niches and protect oxygen-sensitive denitrification enzymes in HNAD bacteria [155,156]. Overall, DO regulates both HN and AD processes by influencing oxygen availability and electron allocation. Effective DO control should therefore accommodate strain-specific physiological characteristics to support aerobic respiration and ammonia oxidation as well as maintaining sufficient electron availability for denitrification.

#### 4.2.4. Interactive Effects of the Environmental Factors

The nitrogen source serves as a critical nexus linking carbon availability, oxygen supply, and the effects of pH and temperature. Assimilation of nitrate or nitrite imposes a higher reducing-equivalent demand than ammonium assimilation [157], thereby rendering the C/N response partly contingent on the nitrogen substrate. Given that reducing equivalents derived from carbon metabolism are also utilized by aerobic respiration, an elevated DO level may impose a constraint on denitrification when carbon availability is limiting [105,153]. The nitrogen source further governs the relevance of pH-dependent speciation: elevated pH and temperature raise the free ammonia fraction in ammonium-containing media, whereas reduced pH increases the free nitrous acid fraction in the presence of nitrite [98,158]. Thus, nitrogen removal reflects both the availability of electrons for the relevant pathways and the chemical forms of nitrogen under the cultivation conditions. Operating optima should therefore be evaluated for specific combinations of nitrogen source, C/N, DO, pH, and temperature.

### 4.3. Effects of Extreme Conditions on HNAD Bacteria

Extreme environmental conditions, particularly high salinity and heavy metal stress, can significantly affect the nitrogen removal performance of HNAD bacteria. Salinity imposes osmotic stress that disrupts cellular metabolism, whereas heavy metals may inhibit key enzymes involved in nitrogen transformation. Nevertheless, several HNAD strains have been reported to sustain efficient nitrogen removal activity under high salinity and heavy metal stress conditions as summarized in Table 6.

#### 4.3.1. Effect of High Salt on HNAD Bacteria

High-salinity wastewater, typically defined as wastewater containing NaCl concentrations exceeding 3% (*w*/*v*) [162], is widely generated from industrial processes such as petroleum exploitation, marine aquaculture, food processing, pharmaceutical manufacturing, and landfill leachate concentration processes [163,164]. Elevated salt concentrations could impose osmotic stress on microbial cells, which can lead to cellular dehydration, disruption of membrane integrity, and reduced enzyme activity [165]. For HNAD bacteria, salinity stress significantly affected both HN and AD processes through alterations in cellular metabolism and osmotic balance [166]. Under high salinity conditions, the increase in extracellular osmotic pressure can cause water efflux through cell membrane, resulting in reduced metabolic activity and slower growth rates [167]. In addition, salinity might suppress the activity of key enzymes such as ammonia monooxygenase (AMO) and nitrate reductase (NR), thereby limiting nitrogen conversion efficiency [166].

Despite the inhibitory effects, a number of HNAD bacteria have demonstrated remarkable tolerance potentials under saline environments. Several salt-tolerant HNAD strains isolated from marine sediments, saline soils, and aquaculture systems with remarkable nitrogen removal capacity have been identified in recent years [8,89], mainly including genus *Acinetobacter, Halomonas, Zobellella, Bacillus, Pseudomonas, Alcaligenes,* and *Flavobacterium* (Table 6). For instance, *Zobellella endophytica* W14, isolated from hypersaline wastewater by Hu et al., was able to grow and efficiently remove ammonium in environments with salinity ranging from 0 to 11% (*w*/*v*) [43]. Similarly, Yao et al. reported that *Bacillus cereus* X7 achieved an ammonium removal efficiency exceeding 97.6% at NaCl concentrations ranging from 0 to 40·g L^−1^ [168]. Similar salt-tolerant characteristics have also been observed in HNAD-based microbial communities operating under saline conditions. Zhang et al. have developed a HNAD bacteria-functionalized biocathode for treating high-salinity ammonium-rich wastewater and reported the TN removal efficiency of 92.5% with stable operation over 350 days [169]. Microbial community analysis revealed the enrichment of salt-tolerant electroactive HNAD bacteria, particularly the genus *Vitellibacter*, which suggested that the coexistence of halotolerance and extracellular electron transfer capability contributed to the sustained nitrogen removal performance under saline conditions [169].

Several protective mechanisms have been proposed to explain salt tolerance in HNAD bacteria. Under saline conditions, the accumulation of compatible solutes, such as ectoine, trehalose, glycine betaine, and proline, may support osmotic balance and help stabilize cellular structures and enzymes in salt-tolerant HNAD strains [8,170]. Hu et al. obtained *Halomonas salifodinae* cultures with different salt tolerances after three cultivation cycles at different salinities [171], indicating that tolerance depended on cultivation history. Metabolite analysis suggested that compatible-solute accumulation contributed to this acclimation response [171]. High salinity may also induce the formation of reactive oxygen species (ROS); therefore, enhanced antioxidant defense systems may contribute to alleviating oxidative damage and maintaining cellular metabolism [160]. In addition, regulation of key nitrogen metabolism enzymes, including AMO and NR, may help certain strains sustain active HNAD pathways under saline conditions [172]. For instance, intracellular ectoine concentrations in *Halomonas* sp. B01 increased with rising NaCl concentrations, while superoxide dismutase (SOD) activity increased significantly at 120 g L^−1^ NaCl [160]. These observations suggest that osmotic regulation and antioxidant responses may contribute to salt tolerance in this strain.

#### 4.3.2. Effect of Heavy Metals on HNAD Bacteria

Heavy metal contamination is another important environmental stressor. Heavy metals such as Cu, Zn, Cr, and Ni are commonly present in industrial effluents generated by metallurgy, electroplating, printing and dyeing, and tanning industries [88]. These metals may bind to cellular proteins and enzymes, disrupt membrane permeability, and interfere with electron transport chains, thereby affecting microbial metabolism [173]. In addition, heavy metals may directly inhibit key enzyme activities involved in nitrogen transformation. Enzymes responsible for aerobic denitrification, including NR and nitrite reductase, can be inhibited through metal binding to active sites or through the generation of reactive oxygen species. As a result, the overall nitrogen removal efficiency may decline under elevated metal concentrations.

Nevertheless, certain HNAD bacteria have demonstrated notable tolerance to heavy metal stress and can maintain efficient nitrogen removal performance in metal-contaminated environments, as summarized in Table 6. For example, Pseudomonas aeruginosa P-1 was reported to achieve an ammonia removal efficiency above 80% at Cr^6+^ concentration of 50.0 mg·L^−1^, with negligible nitrite accumulation [161]. Similarly, *Pseudomonas putida* ZN1 was observed to obtain average NH_4_^+^-N removal efficiency of 94.9% and 92.1% under 60 mg·L^−1^ Cr^6+^ and 8.0 mg·L^−1^ Cu^2+^ stress conditions, respectively [88].

Several protective mechanisms have been proposed to explain heavy metal tolerance in HNAD bacteria. Extracellular polymeric substances (EPS) can adsorb and immobilize metal ions, thereby reducing their direct toxicity to cells [36]. In addition, metal resistance systems, detoxification pathways, and antioxidant defenses may help alleviate metal-induced stress and maintain cellular homeostasis [174]. However, their contribution to nitrogen removal during stress exposure and recovery remains to be further elucidated for individual strains. Moreover, current insights into salinity and metal tolerance may be insufficient to fully determine the rate and extent to which nitrogen transformation activity recovers after stress relief, or whether prior exposure alters subsequent stress responses. These uncertainties may currently constrain predictions of treatment stability under fluctuating conditions.

## 5. Nitrogen Metabolic Mechanism of HNAD Bacteria

### 5.1. Nitrogen Transformation Pathways

Enzymes implicated in reported or proposed HNAD pathways mainly include AMO, hydroxylamine oxidase (HAO), nitrite oxidoreductase (NXR), nitrate reductases, NirK/NirS, Nor, and NosZ. However, their occurrence and functional characterization vary among strains. Direct biochemical evidence, including protein purification, has been obtained for AMO and hydroxylamine oxidase in selected heterotrophic nitrifiers. For example, an active ammonia monooxygenase was purified from *Paracoccus denitrificans* [175], whereas a hydroxylamine oxidase distinct from that of autotrophic nitrifiers was purified from *Thiosphaera pantotropha* [13]. The biochemical basis of ammonia and hydroxylamine oxidation is not yet fully understood across HNAD bacteria, with enzyme assignments in many strains based on genomic annotation or indirect physiological evidence [10]. Similarly, nitrate reduction may involve membrane-bound nitrate reductase (Nar), periplasmic nitrate reductase (Nap), or assimilatory nitrate reductase (Nas), depending on the organism and physiological context. Nas is generally considered to participate in nitrogen assimilation and is therefore distinguished from nitrate reductases associated with denitrification [157].

During nitrogen metabolism, nitrogen may occur as NH_4_^+^, NO_2_^−^, NO_3_^−^, NH_2_OH, NO, N_2_O and N_2_ [8]. Reported HNAD pathways can be broadly grouped into two representative categories. One involves coupled nitrification and denitrification through NO_2_^−^/NO_3_^−^ intermediates [34,126]. The other comprises proposed NH_2_OH-mediated routes to gaseous nitrogen products without NO_3_^−^ or NO_2_^−^ as obligatory intermediates [176,177,178]. These categories reflect the transformation patterns currently reported, although the underlying reactions and enzymes have not yet been equally well resolved across strains. In addition to these pathways, nitrogen assimilation contributes to ammonium removal through incorporation into biomass. Reported or proposed HNAD pathways, representative strains, and their ammonium removal efficiencies are summarized in Table 7.

#### 5.1.1. NO_3_^−^/NO_2_^−^-Mediated Pathways

Wehrfritz et al. proposed a model linking heterotrophic nitrification to aerobic denitrification through shared electron-transfer components in 1993 [13]. A proposed NO_3_^−^/NO_2_^−^ -mediated pathway is illustrated in Figure 3, in which ammonium is oxidized to nitrite via hydroxylamine, with subsequent oxidation to nitrate in some strains. AMO and HAO have been reported in *T. pantotropha* [45], while NXR activity has been reported in *Glutamicibacter arilaitensis* EM-H8 [189]. These observations provide biochemical context for the model rather than verification of the complete pathway within a single strain. In the denitrification module, NO_3_^−^ is reduced to NO_2_^−^ by a dissimilatory nitrate reductase, such as Nap or Nar, depending on the strain and environmental conditions (Equation (1)). NO-forming NirK/NirS subsequently reduce NO_2_^−^ to NO (Equation (2)), Nor reduces NO to N_2_O (Equation (3)), and NosZ catalyzes the reduction of N_2_O to N_2_ (Equation (4)) [190,191]. When all these denitrification steps occur in coupling with the upstream nitrification steps, the overall nitrogen removal sequence can be represented as NH_4_^+^ → NH_2_OH → NO_2_^−^ → NO_3_^−^ → NO_2_^−^→ NO → N_2_O→N_2_. This integrated sequence provides a framework for the reported HNAD pathways, although the specific enzymes and intermediates may vary among strains.

NO_3_^−^/NO_2_^−^ -mediated pathways have been reported or proposed for several HNAD strains exhibiting high ammonia removal efficiencies. For example, *Delftia* sp. B7 achieved an NH_4_^+^-N removal efficiency of 98.7 ± 0.2%, and a removal rate of 4.1 ± 0.04 mg N·L^−1^h^−1^ [179]. Nitrogen transformation experiments and genomic analyses supported a proposed NO_3_^−^/NO_2_^−^-mediated pathway in this strain. However, canonical *amoCBA* and *hao* genes were not identified in the available genome annotation; consequently, the genetic and enzymatic basis of the initial ammonia oxidation steps remains to be further elucidated [179].

2NO_3_^−^ + 4H^+^ + 4e^−^ → 2NO_2_^−^ + 2H_2_O (1)

2NO_2_^−^ + 4H^+^ + 2e^−^ → 2NO + 2H_2_O (2)

2NO + 2H^+^ + 2e^−^ → N_2_O + H_2_O (3)

N_2_O + 2H^+^ + 2e^−^ → N_2_ + H_2_O (4)

In addition, variations of the NO_3_^−^/NO_2_^−^-mediated pathways have been reported, differing mainly in the involvement of NO_3_^−^ and the intermediates included in the proposed sequences (Table 7). These variants can be generally grouped into (i) pathways involving NO_3_^−^ formation before denitrification, (ii) pathways in which NO_2_^−^ enters denitrification without prior oxidation to NO_3_^−^, and (iii) abbreviated reported sequences in which intermediates such as NO or N_2_O are not explicitly represented. Importantly, the omission or non-detection of an intermediate might not be taken as evidence that the corresponding biochemical step is bypassed. The observed diversity in nitrogen transformation pathways suggests that nitrogen transformation varies among HNAD strains [8].

#### 5.1.2. Proposed NH_2_OH-Associated Pathways

In the proposed NH_2_OH-associated pathways, NH_2_OH serves as a putative intermediate linking ammonium oxidation to gaseous nitrogen formation. Several NH_2_OH-associated routes involving NO and/or N_2_O have been reported and are summarized in Figure 3, with representative strains listed in Table 7. The proposed routes mainly include NH_4_^+^ → NH_2_OH → NO → N_2_O →N_2,_ NH_4_^+^ → NH_2_OH → N_2_O→N_2_ and NH_4_^+^ → NH_2_OH → N_2_, differing in the intermediates proposed between NH_2_OH and N_2_.

For *Alcaligenes faecalis* TF-1, a route involving NO and N_2_O as intermediates was proposed based on PCR detection of *amoA*, *hao*, *norB*, and *nosZ*, together with substrate-utilization experiments, which exhibited ammonium removal but no detectable aerobic denitrification when nitrate or nitrite was supplied as the sole nitrogen source [74]. These results support a proposed route but do not, by themselves, establish NH_2_OH, NO, or N_2_O as intermediates or demonstrate the activities of the corresponding enzymes. In *Photobacterium* sp. NNA4, NH_2_OH consumption accompanied by N_2_O production was observed in cell-suspension experiments with uninoculated and heat-inactivated controls, and HAO activity was detected in cell-free extracts. These findings supported the proposed conversion of NH_2_OH to N_2_O. Separate experiments with ^15^N-labeled nitrate and nitrite further demonstrated aerobic production of N_2_O and N_2_ [135], but did not directly establish the complete route from ammonium through NH_2_OH to gaseous nitrogen. For *Corynebacterium glutamicum* WN1, the NH_4_^+^ → NH_2_OH → N_2_ route was proposed by combining NH_2_OH measurements, ^15^NH_4_^+^ tracing, gaseous-product analysis, and genomic and transcriptomic analyses [21]. The direct NH_4_^+^ → NH_2_OH → N_2_ route is proposed but not yet experimentally verified. Trace NH_2_OH and ammonium-derived ^15^N_2_ were detected, and genes annotated as *dnfB* and *dnfC* were upregulated following ammonium addition [21]. However, the absence of detectable NO_2_^−^, NO_3_^−^, or N_2_O does not rule out their involvement in the proposed routes, as rapid formation and consumption may maintain their concentrations below analytical detection limits.

The proposed NH_2_OH-associated pathways may provide alternative routes for nitrogen removal under aerobic conditions. By limiting accumulation of potentially toxic intermediates such as NO_2_^−^ and NO, these pathways may help alleviate cellular stress [93]. Overall, the proposed NH_2_OH-associated pathways highlight the potential diversity of nitrogen metabolism in HNAD bacteria.

#### 5.1.3. Nitrogen Assimilation

While the previous sections primarily focus on dissimilatory pathways that convert inorganic nitrogen into gaseous products, HNAD bacteria can also assimilate inorganic nitrogen into cellular biomass [19,192]. The assimilation process represents an important nitrogen retention mechanism in HNAD bacteria. Therefore, nitrogen transformation in HNAD bacteria is expected to be governed by the balance between assimilatory nitrogen uptake and dissimilatory nitrogen loss. In most bacteria, ammonium assimilation primarily occurs via the glutamine synthetase-glutamate synthase (GS-GOGAT) pathway [193]. The relevant enzymes involved in nitrogen assimilation include glutamine synthase (GS), glutamate synthase (GOGAT) and glutamate dehydrogenase (GDH) [194,195]. In this process, ammonium is first incorporated into glutamine via glutamine synthetase and subsequently converted into glutamate through glutamate synthase. Glutamate then serves as a central nitrogen donor for the biosynthesis of amino acids, nucleotides, and other nitrogen-containing cellular components as illustrated in Figure 3 [91]. Through the assimilatory pathway, inorganic nitrogen can be effectively incorporated into microbial biomass, thereby supporting cellular growth and metabolic activity.

The contribution of nitrogen assimilation varies from different HNAD strains and the representative strains and their reported assimilation contributions are summarized in Table 8. For instance, nitrogen assimilation was the dominant nitrogen removal pathway in *Klebsiella* sp. under aerobic condition [196], as well as *Pseudomonas putida* NP5 can assimilate up to 61.8% of ammonia nitrogen [17]. In contrast, only 24.0% of nitrogen is removed via assimilation in *Rhodococcus* sp. CPZ24 [57]. Therefore, the balance between nitrogen assimilation and dissimilatory nitrogen removal is highly strain-dependent, reflecting the allocation of nitrogen metabolic flux between biosynthetic demand and energy-conserving dissimilatory processes.

The relative contribution of assimilatory nitrogen metabolism in HNAD bacteria is significantly influenced by environmental conditions, particularly the C/N ratio and nitrogen sources. Several studies have suggested that elevated C/N ratios could promote nitrogen assimilation and biomass formation under carbon-sufficient conditions, thereby increasing nitrogen retention within microbial cells [197]. For example, at a C/N ratio of 20, the marine bacterium *Halomonas* sp. DN3 exhibited dominant nitrogen assimilation, with 75.0–85.8% of the removed nitrogen assimilated into cellular biomass [201]. From a metabolic perspective, the assimilatory shift under high C/N ratios is associated with increased availability of carbon skeletons, reducing equivalents (NADH/NADPH), and ATP generated through enhanced carbon oxidation [105]. These metabolic resources might facilitate ammonium incorporation through key assimilation pathways, including the GDH and GS-GOGAT routes, thereby promoting intracellular nitrogen retention rather than extracellular nitrogen transformation [202].

The type of nitrogen source is a critical determinant of assimilatory nitrogen metabolism in HNAD bacteria, owing to differences in energetic requirements and enzymatic regulation. For example, for *Halomonas* sp. B01, nitrogen assimilation accounted for 39.4%, 59.4%, and 56.2% of nitrogen removal when NH_4_^+^-N, NO_3_^−^-N, NO_2_^−^-N were used as the sole nitrogen sources, respectively [160]. Such variation reflects not only strain-specific metabolic characteristics but also the different energetic costs associated with assimilation of reduced and oxidized nitrogen compounds [203]. NH_4_^+^ is generally considered the most energetically favorable nitrogen source because it can be directly incorporated into cellular metabolites without prior reduction [201]. In contrast, the assimilation of NO_3_^−^ and NO_2_^−^ requires additional reduction steps mediated by assimilatory nitrate and nitrite reductases (Nas systems), consuming reducing equivalents and metabolic energy [204]. Under carbon-rich conditions, sufficient electron and energy availability may facilitate these reductive assimilation processes and increase nitrogen retention in biomass.

Finally, assimilatory nitrogen metabolism may enhance the adaptability of HNAD bacteria to environments with elevated ammonium availability. Compared with dissimilatory pathways, ammonium assimilation involves fewer reaction steps, which may facilitate rapid nitrogen incorporation into cellular biomass [40].

### 5.2. The Proposed Nitrogen Metabolic Pathways

The ability of HNAD bacteria to couple HN and AD under aerobic conditions is closely associated with the coordination of carbon metabolism, electron transport processes, and nitrogen transformation pathways [8]. A conceptual framework of this integrated metabolic network is presented in Figure 4, illustrating the proposed distribution of electron flux between oxygen respiration and nitrogen oxide reduction, as well as its linkage to carbon metabolism and enzymatic nitrogen conversions.

During carbon metabolism, organic substrates are oxidized via glycolysis and the tricarboxylic acid (TCA) cycle, generating ATP through aerobic respiration and reducing equivalents (e.g., NADH and FADH_2_) that support cellular metabolism and nitrogen transformation [119]. The intracellular NADH/NAD^+^ ratio reflects cellular redox status and may be associated with changes in respiratory and nitrogen transformation activities [205]. The reducing equivalents subsequently supply electrons (e^−^) to the respiratory ETC, which may link carbon oxidation with nitrogen transformation [206]. Within the ETC, electrons derived from NADH can be partitioned among multiple terminal electron acceptors. Under aerobic conditions, O_2_ typically functions as the primary electron acceptor [129], whereas nitrogen oxides such as NO_3_^−^ and NO_2_^−^ can serve as alternative acceptors in HNAD bacteria [95]. Consequently, reducing equivalents generated through organic carbon oxidation may also support denitrification in the presence of oxygen [93]. The proposed allocation of electrons between oxygen respiration and nitrogen oxide reduction provides a possible explanation for aerobic denitrification (Figure 4). Within this framework, carbon metabolism and electron transport may help coordinate the oxidative reactions of heterotrophic nitrification with the reductive reactions of denitrification.

As described in Section 5.1, enzymes associated with HNAD pathways have been reported in individual strains, although their functional characterization varies. For example, nitrogen balance analysis and enzyme assays in *Paracoccus versutus* LYM supported a proposed pathway linking heterotrophic nitrification with aerobic denitrification [183]. Heterotrophic carbon metabolism provides reducing equivalents that may support both respiratory oxygen reduction and nitrogen oxide reduction. The proposed allocation of reducing equivalents, together with the potential coexistence of enzymes involved in nitrification and denitrification, may support concurrent oxidative and reductive nitrogen transformations under aerobic conditions [105].

## 6. Challenges and Future Perspectives

Although environmental factors, including DO, carbon source type, temperature, salinity, and heavy metal stress, are well recognized as key regulators of HNAD activity, how these factors reshape intracellular redox balance, enzyme activity, and pathway selection remains insufficiently understood. In particular, identifying the conditions that drive shifts in the relative contributions of nitrification, denitrification, and assimilation pathways is essential for developing control strategies to improve system performance and maintain stable operation. The extent and timescale of activity recovery after salinity returns to baseline or inhibitory metals are removed also remain insufficiently characterized in HNAD isolates. Controlled exposure-recovery and re-exposure experiments are needed to assess recovery kinetics, prior exposure effects, and potential irreversible activity loss.

In addition, nitrogen transformation in HNAD bacteria is intimately coupled with carbon metabolism and electron transport. Nevertheless, how electrons are allocated among biomass synthesis, respiratory energy conservation, and nitrogen conversion pathways remains poorly quantified, especially under variable environmental conditions. To address this knowledge gap, future studies should couple measurements of substrate utilization kinetics, carbon consumption, oxygen uptake, and key enzyme activities with nitrogen mass balances that encompass dissolved, biomass-associated, and gaseous nitrogen. Such integrated analyses would distinguish assimilatory nitrogen retention from gaseous nitrogen production and help constrain electron allocation among competing metabolic processes.

Furthermore, although the major nitrogen conversion pathways in HNAD systems—encompassing ammonia oxidation, nitrate/nitrite reduction, and assimilation—have been postulated, their enzymatic underpinnings and relative contributions under aerobic conditions remain poorly characterized. The observation of ammonia oxidation activity in certain strains lacking canonical *amo* and *hao* genes suggests the possible involvement of alternative enzymatic systems or unidentified routes. Hence, future studies should prioritize the elucidation of key functional enzymes and intermediate metabolites, especially those involved in the hydroxylamine-mediated pathways. The integration of enzyme activity assays with time-resolved nitrogen transformation rates would facilitate linking biochemical functions to in situ pathway activities under variable environmental conditions.

Finally, although HNAD bacteria have been applied in laboratory-scale treatment of real wastewater, including livestock-farm and aquaculture wastewater using *Pseudomonas stutzeri* AY4 and *Pseudomonas putida* 4-HYK, respectively [90,207], these demonstrations have yet to establish reliable full-scale performance. Wider application requires addressing carbon and aeration demands, population stability under fluctuating conditions, and potential nitrite accumulation or N_2_O emissions. For example, although HNAD can simplify treatment by integrating nitrogen and COD removal in a single aerobic system compared with conventional nitrification-denitrification [90], realizing this advantage requires optimization of C/N ratio and DO to sustain nitrogen removal rates without excessive carbon inputs or aeration [208]. In addition, key functional enzymes in HNAD, such as nitrate reductase, are oxygen-sensitive, and DO levels strongly affect nitrogen removal and N_2_O emissions [209]. Compared with anaerobic denitrification processes, HNAD exhibits faster growth and greater environmental adaptability, particularly in high-organic-strength wastewater; however, competition for carbon sources among functional microbial groups and N_2_O release have yet to be fully resolved [210]. Furthermore, although the kinetics of pure HNAD strains can be described by Monod or Haldane models, the kinetic parameters of HNAD and the microbial community stability in pilot-scale or full-scale treatment systems still need to be further addressed, particularly the specific nitrogen removal rate and yield coefficients of HNAD bacteria. Overall, HNAD shows considerable promise for treating wastewater with a high C/N ratio in single-reactor systems under energy-efficient operation. Nevertheless, further studies are required to address strain screening, process control, kinetic modeling, N_2_O emission mitigation, and the long-term stability of both strains and microbial communities.

## 7. Conclusions

HNAD bacteria constitute a diverse and functionally distinct microbial guild capable of simultaneous HN and AD, with potential for biological nitrogen removal in wastewater treatment. This review systematically summarizes the current understanding of HNAD bacteria from the perspectives of distribution, physiological characteristics, environmental responses, nitrogen transformation pathways, and metabolic mechanisms. Bacterial isolates reported to exhibit HNAD activity have been successfully isolated from diverse natural and engineered environments, exhibiting considerable physiological versatility across strains. The nitrogen removal performance of HNAD bacteria is governed by the interplay between nitrogen sources, carbon sources, C/N ratio, and environmental conditions (e.g., temperature, pH, dissolved oxygen, salinity, and heavy metals). Reported nitrogen transformation routes include NO_3_^−^/NO_2_^−^-mediated pathways, NH_2_OH-associated pathways, and nitrogen assimilation. Assimilation can contribute substantially to inorganic nitrogen removal through biomass incorporation, and the fate of nitrogen is governed by the balance between dissimilatory transformation and assimilation. Collectively, the physiological versatility and metabolic flexibility of HNAD bacteria highlight their potential for application in sustainable biological nitrogen removal systems. Future research should quantify assimilatory and dissimilatory contributions through comprehensive nitrogen mass balances, identify key enzymes or genes involved, elucidate electron allocation mechanisms, and validate long-term treatment performance under conditions representative of real wastewater.

## Figures and Tables

**Figure 1:**
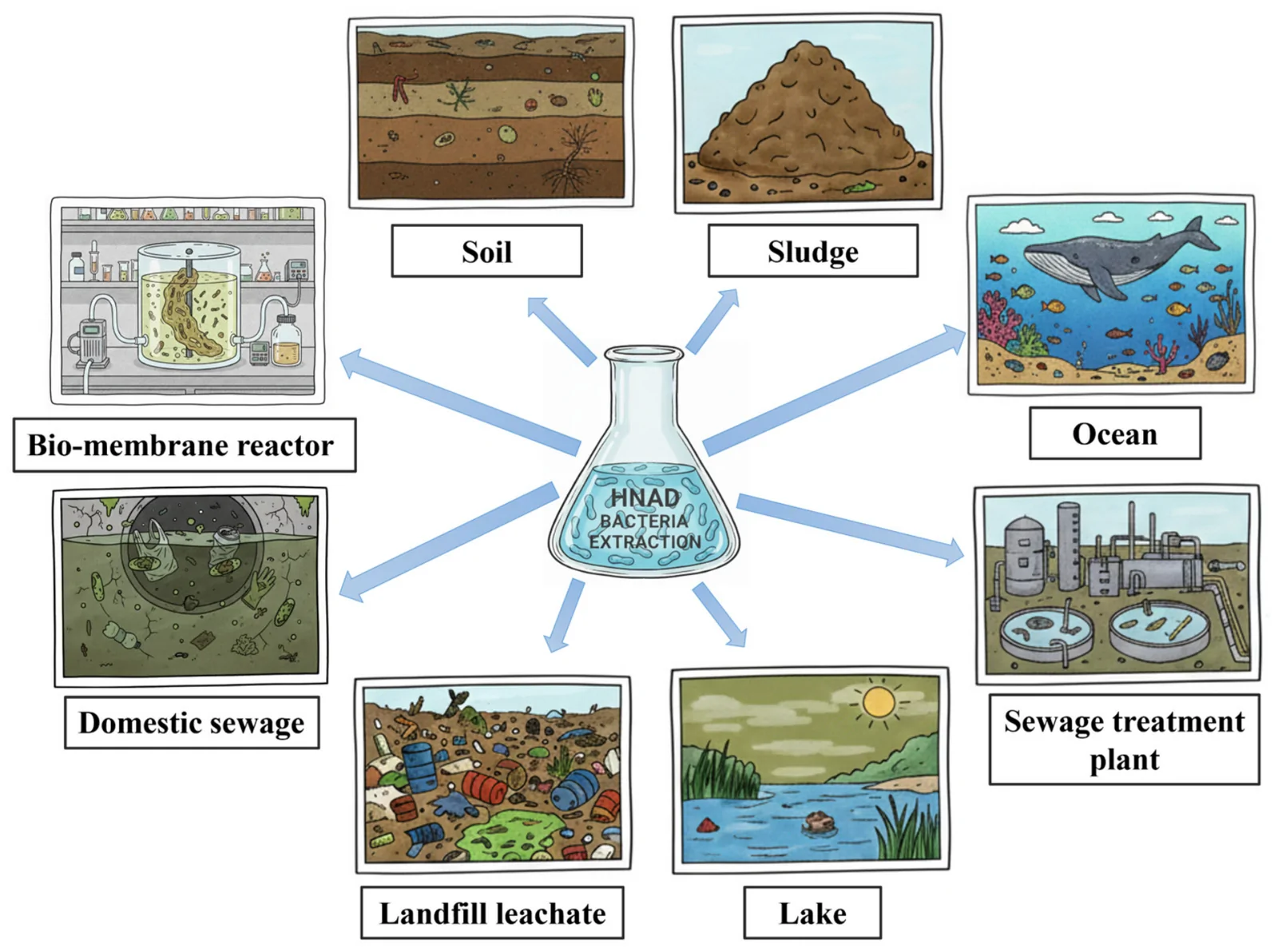
Distribution of isolation environments of HNAD bacteria.

**Figure 2:**
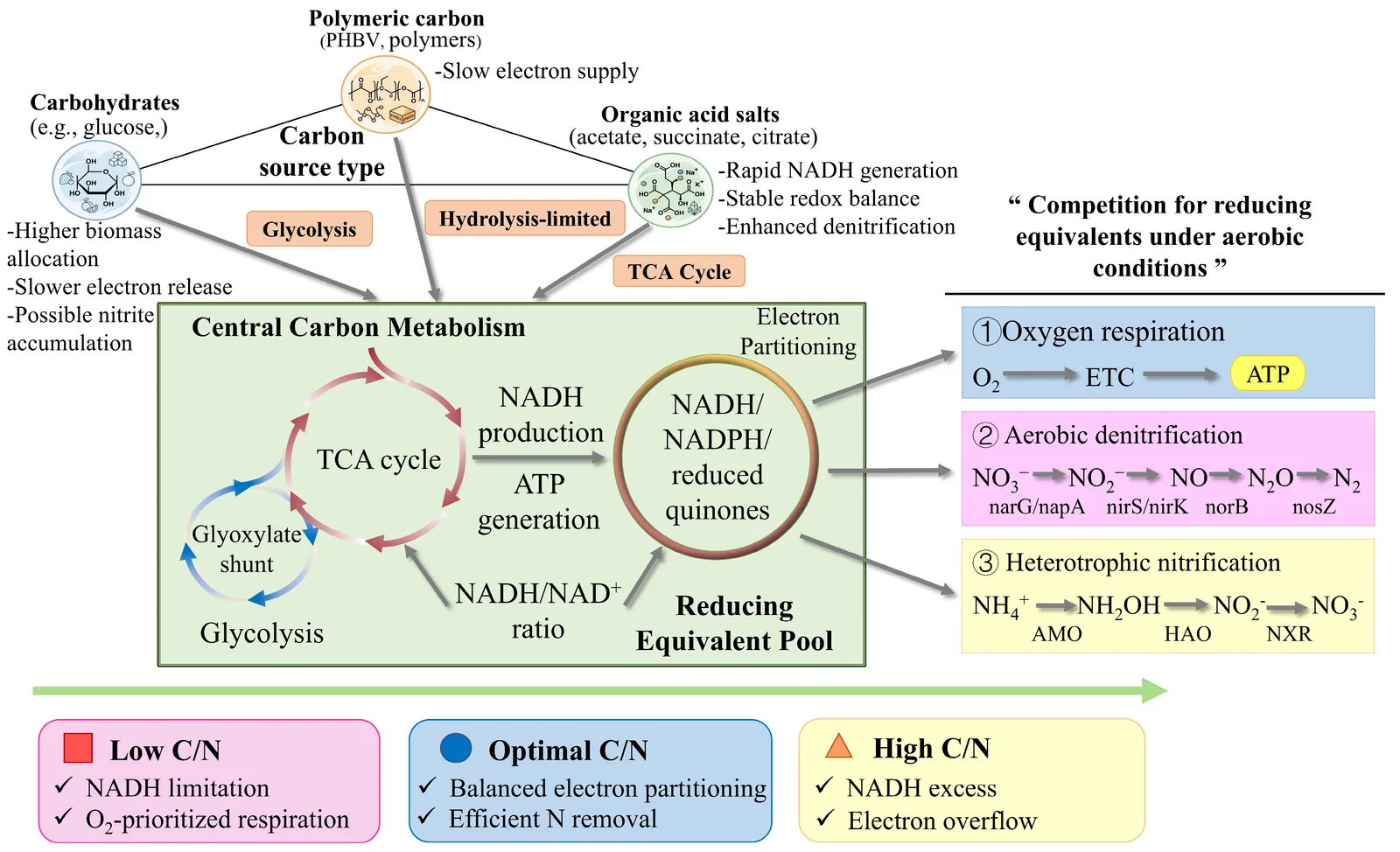
Conceptual framework illustrating carbon source and C/N ratio-regulated electron partitioning in HNAD bacteria.

**Figure 3:**
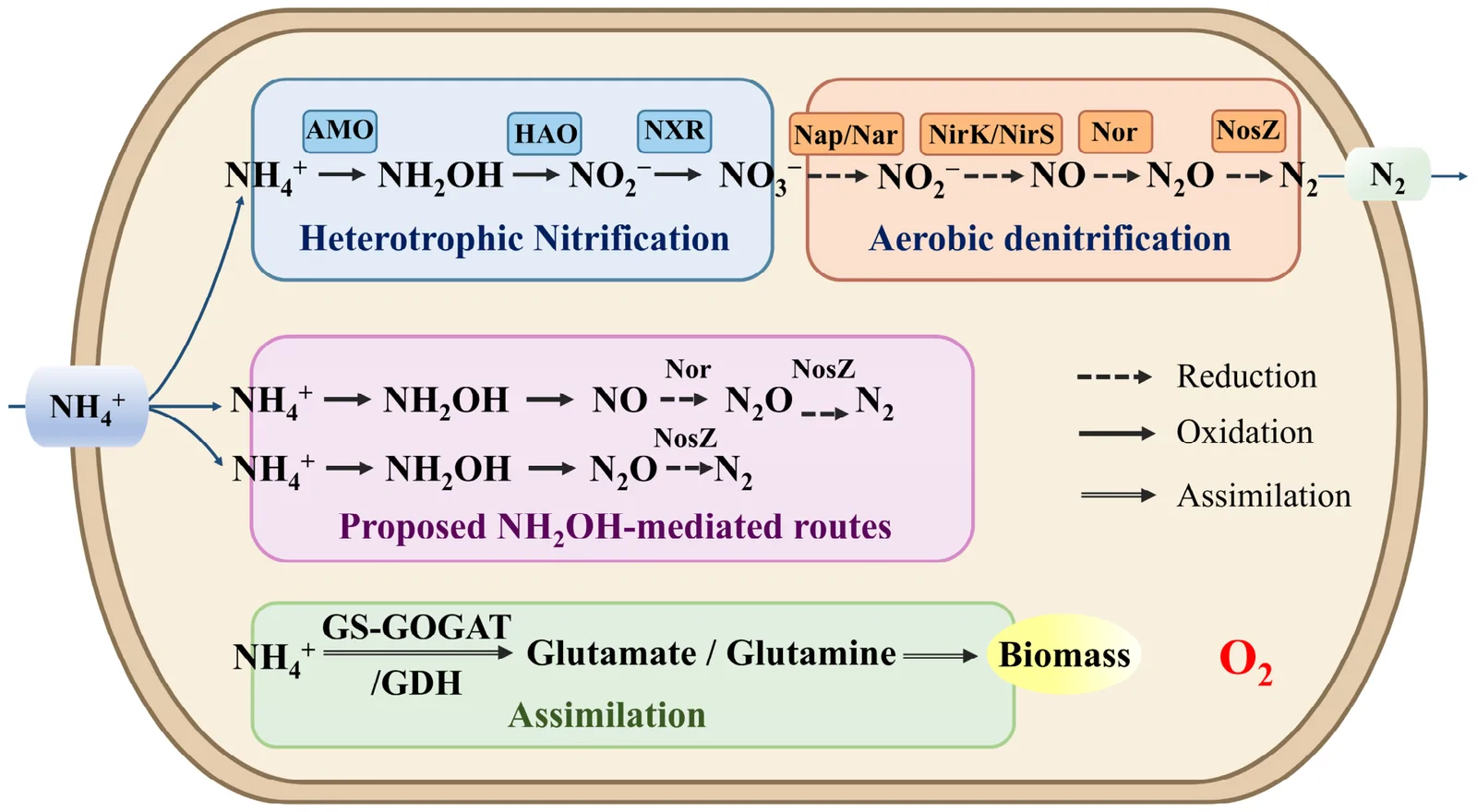
Schematic overview of reported and proposed nitrogen transformation pathways in HNAD bacteria.

**Figure 4:**
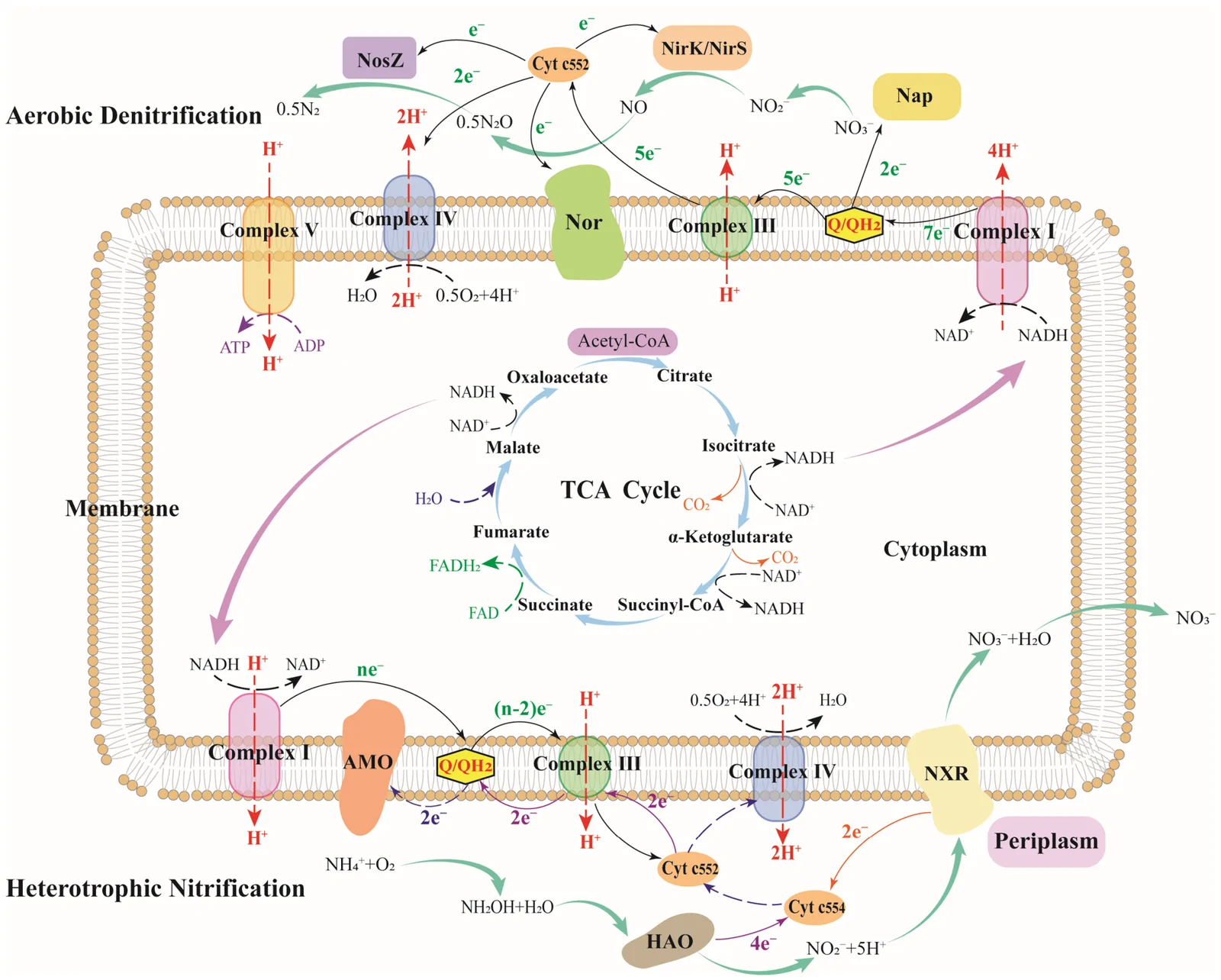
Proposed mechanistic model of nitrogen metabolism in HNAD bacteria.

**Table 1:** Representative HNAD-capable bacterial isolates, their isolation sources, and the methods used to assess their nitrogen-transforming activities.

Genus	Representative Strain	Isolation Source	Nitrogen and Carbon Source	Enrichment Conditions	Methods for Assessing HNAD Activity	Reference
*Pseudomonas*	*Pseudomonas putida* strain LX1	Activated sludge	NH_4_^+^-N; sodium succinate hexahydrate	30 °C; 160 rpm	Aerobic NH_4_^+^/NO_3_^−^-N removal assays; AMO and HAO activity assays	[36]
*Acinetobacter*	*Acinetobacter oleivorans* AHP123	Activated sludge	NH_4_^+^-N; sodium succinate	30 °C; 200 rpm; pH 7.3; C/N 20	NH_4_^+^/NO_3_^−^ removal assays; nitrogen balance; functional gene annotation and RT-qPCR	[37]
*Bacillus*	*Bacillus* sp. L2	Aeration tank water samples	NH_4_^+^-N; sodium acetate	30 °C; 160 rpm; pH 7.0	NH_4_^+^/NO_3_^−^/NO_2_^−^ removal assays; PCR amplification of HNAD-related genes	[38]
*Klebsiella*	*Klebsiella* sp. TN-10	Tannery wastewater	NH_4_^+^-N; sucrose	30 °C; 150 rpm; pH 7.0	NH_4_^+^/NO_3_^−^/NO_2_^−^ removal assays; TN monitoring; AMO, nitrate reductase and Nir activity assays	[39]
*Halomonas*	*Halomonas venusta* SND-01	Marine sediment	NH_4_^+^-N; sodium citrate	30 °C; 120 rpm; pH 7.5–8.5; NaCl (30 g/L)	NH_4_^+^/NO_3_^−^/NH_2_OH/NO_2_^−^ removal assays; genome annotation; AMO, HAO, Nir and Nar activity assays	[40]
*Alcaligenes*	*Alcaligenes ammonioxydans* sp. HO-1	Wastewater treatment sludge	Not reported	Not reported	NH_4_^+^/NO_2_^−^ conversion assays; gaseous product analysis; ^15^N tracing; denitrification inhibition assay; functional cloning of *dnf* gene	[41]
*Vibrio*	*Vibrio* sp. LY1024	Not report	NH_4_^+^-N and NO_2_^−^-N; sodium citrate	pH 7.2	NH_4_^+^/NO_3_^−^/NO_2_^−^ removal assays; nitrogen intermediate monitoring	[42]
*Zobellella*	*Zobellella endophytica* W14	Soil of Ulansuhai Wetland	NO_3_^−^-N; sodium succinate	30 °C, 150 rpm; pH 7.0–7.2; NaCl (50 g/L)	Nitrogen removal assays; nitrogen balance; PCR amplification of *hao*, *napA*, *nirS* and *nosZ*	[43]
*Gordonia*	*Gordonia amicalis* UFV4.	Activated sludge	Not reported	Not reported	NH_4_^+^ removal assays; gaseous product analysis; nitrogen balance	[44]
*Cupriavidus*	*Cupriavidus* sp. S1	Coking wastewater	NH_4_^+^-N; sodium pyruvate	pH 7.0; 30 °C; 120 rpm	NH_4_^+^/NO_3_^−^/NO_2_^−^ removal assays; TN monitoring; N_2_ detection	[30]
*Thiosphaera*	*Thiosphaera pantotropha* LMD 82.5	Sulphide-oxidizing–denitrifying treatment system	NH_4_^+^-N and NO_3_^−^-N; acetate and thiosulfate	pH 8.0; 37 °C; dilution rate 0.03 h^−1^	Nitrogen balance; O_2_ uptake assays; AMO and HAO activity assays	[45]
*Aeromonas*	*Aeromonas* sp. HN-02	Activated sludge	Not reported	Not reported	NH_4_^+^/NO_3_^−^/NO_2_^−^ removal assays; TN monitoring	[46]
*Providencia*	*Providencia rettgeri* YL	MBR reactor	Peptone and beef extract	NaCl; 30 °C; 120 rpm; 24 h	^15^N tracing; N_2_/N_2_O isotope analysis; nitrogen balance	[47]
*Serratia*	*Serratia* sp. LJ-1	Membrane bioreactor	NH_4_^+^-N; glucose and peptone	pH 7.0–8.0; 30 ± 2 °C 100 rpm	NH_4_^+^ removal and NO_3_^−^/NO_2_^−^ production assays; nitrogen-species monitoring	[48]
*Streptomyces*	*Streptomyces mediolani* EM-B2	Not reported	Not reported	Not reported	Nitrogen removal assays; nitrogen balance; inhibition assays; AMO, HAO, NR and Nir activity assays	[49]
*Diaphorobacter*	*Diaphorobacter* sp. PD-7	Activated sludge	NH_4_^+^-N; phenol	pH 7.2–7.4; 30 °C; 180 rpm	NH_4_^+^/NO_3_^−^ removal assays; nitrogen intermediate monitoring; HAO, Nar and Nir activity assays	[50]
*Delftia*	*Delftia tsuruhatensis* SDU2	Waste treatment pond of pig farm	Acetamide	30 °C	NH_4_^+^/NO_3_^−^/NO_2_^−^ removal assays; TN and biomass-N measurements; PCR amplification and RT-qPCR of HNAD-related functional genes	[51]
*Achromobacter*	*Achromobacter* sp. GAD-3	Sediment and sewage from the Longfeng Wetland	NH_4_^+^-N and NO_3_^−^-N; sodium acetate	pH 7.0; 30 °C; 150 rpm	Nitrogen-species monitoring; gaseous product analysis; nitrogen balance; Nar, Nir, Nor and N_2_or activity assays; RT-qPCR	[52]
*Janthinobacterium*	*Janthinobacterium* sp. J1-1	Wastewater	NH_4_^+^-N; sodium succinate	pH 7.0 ± 0.2; 8 °C; 100 rpm	NH_4_^+^/NO_3_^−^/NO_2_^−^ removal assays; TN monitoring; genome annotation; PCR amplification of HNAD-related functional genes	[53]
*Ochrobactrum*	*Ochrobactrum anthropic* LJ81	Domestic sludge	NO_3_^−^-N; sucrose	30 °C; 150 rpm	NH_4_^+^/NO_3_^−^/NO_2_^−^ removal assays; nitrogen balance; chlorate inhibition assays; PCR amplification of denitrification gene	[54]
*Chryseobacterium*	*Chryseobacterium* sp. R31	Abattoir wastewater	NH_4_^+^-N; glucose	pH 6.8–7.5, 120 rpm	NH_4_^+^/NO_3_^−^/NO_2_^−^ removal assays; nitrogen balance estimation; chlorate inhibition; denitrification gene PCR	[55,56]
*Rhodococcus*	*Rhodococcus* sp. CPZ24	Swine wastewater ponds	NH_4_^+^-N; sodium succinate hexahydrate	30 °C; pH 7.2; 120 rpm	NH_4_^+^/NO_3_^−^ removal assays; intracellular-N measurement; N_2_/N_2_O detection	[57]

**Table 2:** Comparison of $\mu_{\max}$ between HNAD bacteria and autotrophic nitrifiers.

Microorganism Type	Representative Strains	Nitrogen Substrate	$\mu_{\max}$ (h^−1^)	Reference
HNAD bacteria	*Alcaligenes faecalis* No. 4	NH_4_^+^-N	0.20	[64]
	*Agrobacterium* sp. LAD9	NH_4_^+^-N	0.32	[78]
	*Achromobacter* sp. GAD3/GAD4		0.31/0.38	
	*Bacillus* spp.	NH_4_^+^-N	0.43–0.55	[65]
	*Paracoccus denitrificans* ISTOD1	NH_4_^+^-N	0.52	[79]
	*Cupriavidus* sp. S1	NH_4_^+^-N	0.186	[30]
	*Klebsiella pneumoniae* CF-S9	NH_4_^+^-N	0.55	[80]
	*Ochrobactrum anthropic* LJ81	NH_4_^+^-N, NO_3_^−^-N, NO_2_^−^-N	0.36, 0.324, 0.315	[54]
	*Pseudomonas stutzeri* KTB	NH_4_^+^-N, NO_3_^−^-N, NO_2_^−^-N	0.56, 0.42, 0.45	[81]
	*Thiosphaera pantotropha*	NH_4_^+^-N, NH_4_^+^-N+NO_3_^−^-N, NO_3_^−^-N	0.28–0.45, 0.34–0.58, 0.25–0.49	[34]
Autotrophic nitrifiers (AOB)	*Nitrosomonas europaea*	NH_4_^+^-N	0.03–0.05	[34]
	*Nitrosomonas mobilis* Ms1	NH_4_^+^-N	0.05–0.07	[82]

$\mu_{\max}$: maximum specific growth rate; Values were reported in the literature and may vary with temperature, substrate type, and cultivation conditions.

**Table 3:** Nitrogen source preference and removal characteristics of representative HNAD bacteria.

Strain	Nitrogen Preference	Nitrogen Removal Rates (mgN·L^−1^·h^−1^) or Efficiency (%)	Nitrogen Transformation Traits	Reference
*Acinetobacter* sp. ND7	NH_4_^+^ > NO_2_^−^ > NO_3_^−^	NH_4_^+^ 5.7; NO_2_^−^ 4.2; NO_3_^−^: 3.6	NO_2_^−^ accumulation	[70]
*Lysinibacillus fusiformis* B301	NH_4_^+^ > NO_3_^−^ > NO_2_^−^	NH_4_^+^ 2.1; NO_3_^−^ 1.6; NO_2_^−^ 1.4	83.25% NH_4_^+^-N to N_2_	[84]
*Halomonas *sp. 5505	NH_4_^+^ prior to NO_3_^−^/NO_2_^−^	NH_4_^+^ 100%; NO_3_^−^ 94.1%; NO_2_^−^ 74.4%	Assimilation-dominated	[85]
*Hydrogenophaga* sp. H7	NH_4_^+^ > NO_3_^−^	NH_4_^+^ 1.4; NO_3_^−^ 0.9;	NH_4_^+^-N removal inhibited by NO_3_^−^/NO_2_^−^ addition	[86]
	NH_4_^+^ > NO_2_^−^	NH_4_^+^ 1.6; NO_2_^−^ 2.3		
*Paracoccus denitrificans* HY-1	NH_4_^+^ prior to NO_3_^−^/NO_2_^−^	NH_4_^+^ 10.7; NO_3_^−^ 22.1; NO_2_^−^ 27.8	NH_4_^+^-N enhanced the removal of NO_3_^−^-N and NO_2_^−^-N	[87]
*Pseudomonas putida* ZN1	NH_4_^+^ prior to NO_3_^−^/NO_2_^−^	NH_4_^+^ 94.5%; NO_3_^−^ 86.1%; NO_2_^−^ 71.6%	Preferential NH_4_^+^-N oxidation followed by denitrification without intermediate accumulation	[88]

**Table 4:** The optimal carbon source and C/N ratio for representative HNAD bacteria.

Strain	Carbon Source	Metabolic Entry Point	Optimal C/N Ratio	Removal Efficiency	Reference
*Pseudomonas aeruginosa* SNDPR-01	sodium citrate	TCA cycle intermediate	10	NH_4_^+^-N 87%	[26]
*Acinetobacter* ZHG-1	sodium citrate	TCA cycle intermediate	30	NH_4_^+^-N 97.1%	[105]
*Paracoccus* LYM	sodium lactate	Pyruvate precursor	15–30	NH_4_^+^-N 97.1%	[106]
*Bacillus* GS-5	sodium acetate	Short-chain fatty acid	7.5	NH_4_^+^-N 95.6%	[107]
*Bacillus* I6	glucose	Carbohydrate	8	NH_4_^+^-N 85%	[108]
*Thauera* sp. *SND5*	Sodium lactate	Pyruvate precursor	7.5	NH_4_^+^-N 99.2%	[109]
*Cupriavidus* S1	sodium pyruvate	Central metabolic intermediate	12–28	NH_4_^+^-N 99.7%	[30]
*Bacillus* LY	glucose	Carbohydrate	15	NH_4_^+^-N 100%	[110]
*Zobellella* sp. B307	sodium succinate	TCA cycle intermediate	5	NH_4_^+^-N 97.7%	[111]
*Pseudomonas putida* S3	sodium citrate	TCA cycle intermediate	6	NH_4_^+^-N 73.3%	[112]
*Pseudomonas stutzeri* AY4	glucose	Carbohydrate	6	NH_4_^+^-N 95.8%	[113]
	sodium succinate	TCA cycle intermediate		NO_3_^−^-N 91.4%	
*Chryseobacterium* R31	glucose	Carbohydrate	10	NH_4_^+^-N 95.9%	[55]
	sodium succinate	TCA cycle intermediate			

**Table 5:** Environmental characteristics and nitrogen removal performance of representative HNAD bacteria.

Strain	Nitrogen Source	Optimal Temperature (°C)	Optimal pH	DO Condition (rpm)	Nitrogen Removal Efficiency	Reference
*Delftia tsuruhatensis* SDU2	NH_4_^+^-N	30	7	150	NH_4_^+^-N 93.7%	[51]
*Janthinobacterium* sp. J1-1	NH_4_^+^-N; NO_3_^−^-N	8	7	150	NH_4_^+^-N 89.2%; NO_3_^−^-N 68.2%	[53]
*Pseudomonas stutzeri* strain AY4	NH_4_^+^-N; NO_3_^−^-N	30	7–8	180	NH_4_^+^-N 92.2%; NO_3_^−^-N 94.6%	[113]
*Pseudomonas peli* NR-5	NH_4_^+^-N; NO_3_^−^-N; NO_2_^−^-N	10	7	144	NH_4_^+^-N 97.3%; NO_3_^−^-N 95.3%; NO_2_^−^-N 87.8%	[130]
*Paracoccus denitrificans* AC-3	NH_4_^+^-N	35	6–10	150	NH_4_^+^-N > 99%	[131]
*Pseudomonas tolaasii* Y-11	NH_4_^+^-N; NO_3_^−^-N; NO_2_^−^-N	15	7.2	150	NH_4_^+^-N 93.6%; NO_3_^−^-N 93.5%; NO_2_^−^-N 81.9%	[27]
*Acinetobacter baumannii* AL-6	NH_4_^+^-N	30	7	120	NH_4_^+^-N 99.8%	[132]
*Pseudomonas mosselii* K17	NH_4_^+^-N	30	7	165	NH_4_^+^-N > 95%	[133]
*Paracoccus denitrificans* XW11	NH_4_^+^-N; NO_3_^−^-N	30	7.2	160	NH_4_^+^-N 100%; NO_3_^−^-N 90.5%	[129]
*Acinetobacter* ZHG-1	NH_4_^+^-N	30	9	150	NH_4_^+^-N 97.1%	[105]
*Bacillus* GS-5	NH_4_^+^-N	35	7.5	125	NH_4_^+^-N 95.6%	[107]
*Chryseobacterium* R31	NH_4_^+^-N	30	7	120	NH_4_^+^-N 99.7%	[55]
*Vibrio diabolicus* SF16	NH_4_^+^-N	30	7.2	120	NH_4_^+^-N 97.1%	[58]
*Janthinobacterium* sp. M-11	NH_4_^+^-N; NO_3_^−^-N; NO_2_^−^-N	2	7	150	NH_4_^+^-N 98%; NO_3_^−^-N 89%; NO_2_^−^-N 89%	[134]

**Table 6:** Nitrogen removal performance of HNAD bacteria under high salinity and heavy metal stress.

Stress Condition	Strain	Stress Level	Nitrogen Removal Efficiency	Reference
High salinity	*Zobellella endophytica* W14	5% NaCl	NH_4_^+^-N 100%; NO_3_^−^-N 98.9%; NO_2_^−^-N 99.6%	[43]
	*Halomonas* sp. 5505	8% NaCl	NH_4_^+^-N 100%; NO_3_^−^-N 94.1%; NO_2_^−^-N 74.4%	[85]
	*Acinetobacter* sp. JR1	20 g·L^−1^ NaCl	NH_4_^+^-N 96.0%	[144]
	*Serratia marcescens* CL1502	1–5% NaCl	NH_4_^+^-N > 90.0%	[159]
	*Zobellella* sp. B307	75 g·L^−1^ NaCl	NH_4_^+^-N 97.7%; NO_3_^−^-N 94.4%	[111]
	*Halomonas* sp. B01	60 g·L^−1^ NaCl	NH_4_^+^-N 98.8%	[160]
	*Bacillus* N31	30–40‰ NaCl	NH_4_^+^-N > 80.17%	[71]
	*Pseudomonas aeruginosa* P-1	40 g·L^−1^ NaCl	NH_4_^+^-N 100%	[161]
heavy metals	*Pseudomonas putida* LX1	25.0 mg·L^−1^ Cr^6+^	NH_4_^+^-N 83.4%	[36]
	*Aeromonas* sp. HN-02	8.0 mg·L^−1^ Zn^2+^; 0.5 mg·L^−1^ Cu^2+^ + Zn^2+^	NH_4_^+^-N 94.6%; NH_4_^+^-N 46.4%	[46]
	*Acinetobacter* sp. JR1	20.0 mg·L^−1^ Mn^2+^	NH_4_^+^-N 90.5%	[144]
	*Pseudomonas putida* NP5	50.0 mg·L^−1^ Cr^6+^	NH_4_^+^-N > 40%	[17]
	*Pseudomonas putida* ZN1	60.0 mg·L^−1^ Cr^6+^; 20.0 mg·L^−1^–80.0 mg·L^−1^ Ni^2+^	NH_4_^+^-N 94.9%; NH_4_^+^-N 71.3–31.5%	[88]
	*Pseudomonas aeruginosa* P-1	30 mg·L^−1–^50 mg·L^−1^ Cu^2+^; 50 mg·L^−1^ Cr^6+^	NH_4_^+^-N 100–52.18%; NH_4_^+^-N > 80%	[161]

**Table 7:** Reported or proposed nitrogen transformation pathways in bacteria described as HNAD strains and their ammonium removal performance.

Pathways	Reported or Proposed Nitrogen Transformation Pathway	Strain	NH_4_^+^-N Removal Efficiency (%)	Reference
NO_3_^−^/NO_2_^−^-mediated pathways	NH_4_^+^ → NH_2_OH → NO_2_^−^ → NO_3_^−^ → NO_2_^−^→ NO → N_2_O→N_2_	*Pseudomonas stutzeri* KTB	NH_4_^+^-N 100%	[81]
		*Paracoccus denitrificans* HY-1	NH_4_^+^-N 98.1%	[120]
		*Providencia rettgeri* YL	NH_4_^+^-N 80–97%	[47]
		*Ochrobactrum anthropic* LJ81	NH_4_^+^-N > 80%	[54]
		*Delftia* sp. B7	NH_4_^+^-N 98.7%	[179]
		*Pseudomonas* sp. JQ-H3	NH_4_^+^-N 95.4%	[180]
	NH_4_^+^ → NH_2_OH → NO_2_^−^ → NO_3_^−^ → NO_2_^−^→ NO → N_2_O	*Pseudomonas* sp. ZHL02	NH_4_^+^-N 99.1%	[93]
	NH_4_^+^ ↔glutamine→ NH_2_OH → NO_2_^−^ → NO_3_^−^ → NO_2_^−^→ NO → N_2_O→N_2_	*Zobellella* sp. An-6	NH_4_^+^-N 96.1%	[181]
	NH_4_^+^ → NH_2_OH → NO_2_^−^ → NO_3_^−^ → NO_2_^−^ → N_2_O→N_2_	*Acinetobacter junii* YB	NH_4_^+^-N 99.9%	[176]
	NH_4_^+^ → NH_2_OH → NO_2_^−^ → NO_3_^−^ → NO_2_^−^→N_2_	*Pseudomonas fluorescens* wsw-1001	NH_4_^+^-N 71.2%	[182]
		*Paracoccus versutus* LYM	NH_4_^+^-N 75.4%	[183]
		*Lysinibacillus fusiformis* B301	NH_4_^+^-N 83.3%	[84]
	NH_4_^+^ → NH_2_OH → NO_2_^−^ → NO_3_^−^→ N_2_O→N_2_	*Bacillus subtilis* A1	NH_4_^+^-N 58.4%	[184]
		*Rhodococcus* sp. CPZ24	NH_4_^+^-N 85%	[57]
		*Klebsiella pneumoniae* CF-S9	NH_4_^+^-N 85%	[80]
	NH_4_^+^ → NH_2_OH → NO_2_^−^ → N_2_O→N_2_	*Agrobacterium* sp. LAD9	NH_4_^+^-N 90.1%	[23]
		*Alcaligenes ammonioxydans* sp. HO-1	NH_4_^+^-N 84%	[41]
	NH_4_^+^ → NH_2_OH → NO_2_^−^ → NO→ N_2_O→N_2_	*Pseudomonas stutzeri* YG-24	NH_4_^+^-N 88.1%	[90]
NH_2_OH-mediated pathways	NH_4_^+^ → NH_2_OH → NO → N_2_O→N_2_	*Alcaligenes faecalis* TF-1	NH_4_^+^-N 94.6%	[74]
	NH_4_^+^ → NH_2_OH → N_2_O→N_2_	*Bacillus methylotrophicus* L7	NH_4_^+^-N 91%	[66]
		*Acinetobacter calcoaceticus* HNR	NH_4_^+^-N 92.3%	[178]
		*Photobacterium* sp. NNA4	NH_4_^+^-N > 99.9%	[135]
		*Alcaligenes faecalis* No. 4	NH_4_^+^-N > 99.9%	[185]
		*Alcaligenes faecalis* NR	NH_4_^+^-N 66.7–78.3%	[186]
	NH_4_^+^ → NH_2_OH →N_2_	*Corynebacterium glutamicum* WN1	NH_4_^+^-N > 99.9%	[21]
Other novel pathways	NH_4_^+^ → NO → N_2_O	*Pseudomonas putida* Y-9	NH_4_^+^-N 99.41%	[187]
	NH_4_^+^ → N_2_O→N_2_	*Pseudomonas taiwanensis* sp. J488	NH_4_^+^-N > 99.9%	[188]

**Table 8:** Contribution of assimilatory nitrogen metabolism in reported HNAD bacteria.

Strain	Nitrogen Source	Assimilation Contribution (%)	Dominant Nitrogen Pathway	Reference
*Bacillus* sp. LY	NH_4_^+^-N	40.5%	Multiple pathways	[110]
*Providencia rettgeri strain* YL	NH_4_^+^-N	49.7%	Multiple pathways	[47]
*Agrobacterium* sp. LAD9	NH_4_^+^-N	40.8%	Multiple pathways	[23]
*Pseudomonas putida* NP5	NH_4_^+^-N	61.8%	Assimilation-dominated	[17]
*Acinetobacter haemolyticus* JS-1	NH_4_^+^-N; NO_3_^−^-N	74.5%; 85.9%	Assimilation-dominated	[197]
*Rhodococcus* sp. CPZ24	NH_4_^+^-N	24%	Multiple pathways	[57]
*Enterobacter asburiae* GS2	NH_4_^+^-N	63.7%	Assimilation-dominated	[198]
*Halomonas* sp. B01	NH_4_^+^-N;	39.4%	Multiple pathways	[160]
*Halophilic Vibrio diabolicus* SF16	NH_4_^+^-N	35.8%	Multiple pathways	[58]
*Alcaligenes faecalis* C16	NH_4_^+^-N	28–45%	Multiple pathways	[199]
*Acinetobacter calcoaceticus* HNR	NH_4_^+^-N	52.1%	Assimilation-dominated	[178]
*Corynebacterium glutamicum* WN_1_	NH_4_^+^-N	62.4%	Assimilation-dominated	[21]
*Ochrobactrum anthropi* HND19	NH_4_^+^-N; NO_3_^−^-N; NH_4_^+^-N + NO_3_^−^-N	37.9%; 26.4%; 27.9%	Multiple pathways	[200]
*Ochrobactrum anthropic* LJ81	NH_4_^+^-N; NO_3_^−^-N; NO_2_^−^-N	9.5%; 10.6%; 11.1%	Multiple pathways	[54]
*Paracoccus denitrificans* HY-1	NH_4_^+^-N	80.7%	Assimilation-dominated	[120]

## Data Availability

No new data were created or analyzed in this study. Data sharing is not applicable to this article.
